# Theranostic digital twins for personalized radiopharmaceutical therapies: Reimagining theranostics *via* computational nuclear oncology

**DOI:** 10.3389/fonc.2022.1062592

**Published:** 2022-12-15

**Authors:** Arman Rahmim, Julia Brosch-Lenz, Ali Fele-Paranj, Fereshteh Yousefirizi, Madjid Soltani, Carlos Uribe, Babak Saboury

**Affiliations:** ^1^ Department of Radiology, University of British Columbia, Vancouver, BC, Canada; ^2^ Department of Integrative Oncology, BC Cancer Research Institute, Vancouver, BC, Canada; ^3^ School of Biomedical Engineering, University of British Columbia, Vancouver, BC, Canada; ^4^ Department of Electrical and Computer Engineering, University of Waterloo, Waterloo, ON, Canada; ^5^ Department of Functional Imaging, BC Cancer, Vancouver, BC, Canada; ^6^ Department of Radiology and Imaging Sciences, Clinical Center, National Institutes of Health, Bethesda, MD, United States

**Keywords:** digitals twins, theranostics, personalized medicine, radiopharmaceutical therapies, molecular imaging

## Abstract

This work emphasizes that patient data, including images, are not operable (clinically), but that digital twins are. Based on the former, the latter can be created. Subsequently, virtual clinical operations can be performed towards selection of optimal therapies. Digital twins are beginning to emerge in the field of medicine. We suggest that theranostic digital twins (TDTs) are amongst the most natural and feasible flavors of digitals twins. We elaborate on the importance of TDTs in a future where ‘one-size-fits-all’ therapeutic schemes, as prevalent nowadays, are transcended in radiopharmaceutical therapies (RPTs). Personalized RPTs will be deployed, including optimized intervention parameters. Examples include optimization of injected radioactivities, sites of injection, injection intervals and profiles, and combination therapies. Multi-modal multi-scale images, combined with other data and aided by artificial intelligence (AI) techniques, will be utilized towards routine digital twinning of our patients, and will enable improved deliveries of RPTs and overall healthcare.

## Introduction

Patient data, including images, are not the end, but only the beginning of a complex path. They provide the basis for healthcare providers and clinicians to diagnose diseases and to make wise treatment and care decisions for patients. This involves thought-experiments by clinicians: if I pursue treatment plan A, what will happen? What if I prescribe treatment plan B? What about plan C, and so on. Digital twins are virtual avatars of individual patients designed precisely to aid with that. They are not merely images. They embody biological and physiological relationships. They will not fit existing DICOM formats, as we are used to for images. This is not about manipulating images as we are used to with existing powerful software, but about experimenting with digital versions of our patients, i.e. virtual therapies, to answer questions about therapy effects and to propose optimal treatments for individual patients.

Digital twins have been used extensively in engineering for years, and oncology is just now at the cusp of utilizing them (1). In the original industrial context, digital twins have involved use of mathematical and computational models to virtually represent a physical object, predict its behavior, and facilitate decision-making to improve that behavior in the future (2). As an example, digital twinning was defined by NASA in 2010 as an “integrated multi-physics, multi-scale, probabilistic simulation of a vehicle or system that uses the best available physical models, sensor updates, etc., to mirror the life of its flying twin” (3). More recently, digital twins have been used to refer to methods that can collect large datasets with accurate mathematical models to characterize important aspects of the spatial and temporal dynamics of the phenomena being studied.

The idea of creating and using digital twins to encapsulate patient dynamics and to individualize the care of patients has grown in popularity. More specifically for oncology, this is related to improvements in experimental techniques for quantitatively characterizing cancer as well as improvements in the mathematical and computational sciences (2). These include development of tissue-scale models for 1) identifying pathophysiological characteristics of tumors (4), 2) predicting spatiotemporal changes of tumor size, shape, tumor cell density, and response to administered therapies (5, 6), and 3) identifying and optimizing treatment options on a patient-specific basis. These have created significant interest in developing and utilizing image-guided digital twins for clinical oncology (7). Medical imaging data from procedures such as x-ray CT, MRI and PET, can provide longitudinal *in vivo* measurements of cancers of individuals, and can be used to inform modeling at the tissue scale.

Building a digital twin starts with a blueprint of human biology (digital template) and then integrates all that we know about a patient during one’s lifetime (personalization). It can be updated with real-time data (e.g. patient’s overall health condition and existing diseases, diagnostic imaging, histopathologic results from biopsies as well as pre- and intra-therapeutic imaging or cumulative absorbed radiation doses) reflecting history and current condition of the patient. It will have different versions, representing the patient’s evolution at different points in time. Importantly, it can be used for virtual simulation of interventions and to predict their effects to aid physicians in complex treatment planning scenarios towards improved personalized therapies.

A patient’s digital twin can be created using advanced biomedical imaging, which includes patient-specific measurements of tumor growth and response as well as noninvasive, serial observations of the patient’s physical state’s spatiotemporal variations. Medical imaging information gathered from patients can be used to initialize and customize mathematical models. The image-guided mathematical model parameters enable personalized digital representations of patient disease and tumor characteristics, allowing for forecasting of treatment and patient response. The objective is to improve treatment outcomes for specific patients, by developing patient-individual models to simulate disease progression and treatment outcomes as well as to pinpoint mechanistic explanations for patients’ varying responses to treatment (8, 9).

There are increasing interests and efforts in the space of digital twinning of patients (10, 1). And we believe that amongst the most natural, feasible and on-the-near-immediate-horizon approaches to digital twins are so-called theranostic digital twins (TDTs), which we describe next.

## Theranostic digital twins

Theranostics in nuclear medicine is a rapidly emerging field of practice, in which the same target is used for molecular imaging as well as molecular targeted radiopharmaceutical therapies (RPTs) (11). As an example, if cancer cells in a patient express a particular receptor, and we design a molecule to bind to that target, then radiolabeling such a molecule with an isotope that decays *via* the emission of gamma rays or positrons (that lead to gamma rays) enables molecular imaging, while radiolabeling with an isotope that emits particles (e.g. alpha or beta particles, or Auger electrons) enables RPTs.

Theranostic clinical trials have shown notable success [e.g. (12–14)]. However, the existing one-size-fits-all paradigm for treatment activity is suboptimal (15), and arguably unethical. Each RPT cycle typically consists of injecting fixed radioactivities of the radiopharmaceutical to all patients undergoing therapy (e.g. 200mCi for Lu-177-PSMA and Lu-177-DOTATATE). In other words, molecular imaging is presently used only to *identify* which patients can benefit from RPTs, and *not* to optimize their therapies. In fact, the range of uncertainties in absorbed doses delivered to organs-at-risk (max:min) can easily span 1 order of magnitude (16, 17). By injecting our patients with the same radioactivities independent of patient differences (such as pre-treatments, tumor burden, weight, height, etc.), we are all but guaranteeing for them to receive *different* absorbed doses!

The effect of RPTs on tumors and normal tissues is through absorbed dose (dose-effect paradigms). Variation in absorbed doses is highly uncontrolled at the moment but can be predicted and managed. That is the irony of fixed administered activity. Fixed administered activity and unknown variations in absorbed dose have led to conservative paradigms in which many patients are potentially being undertreated and possible dose-toxicity relationships remain unrevealed. We can do better for our patients. We should do better for our patients. And TDTs have a significant role to play to overcome the limitation of non-personalized RPTs.

We need to measure absorbed doses delivered to our patients. More importantly, we need to *predict* absorbed doses delivered to our patients, and we are commonly doing neither! In *retrospective* dosimetry, we can achieve the former; e.g. Lu-177 radiolabeled molecules which are used for RPTs also emit gamma rays that enable utilization of quantitative SPECT/CT imaging to estimate absorbed doses delivered to tumors and organs-at-risk. But can we also do *predictive* dosimetry? Can we use a pre-therapy PET/CT scan and/or intra-therapy SPECT/CT scan(s) to predict absorbed doses delivered in future cycles of RPTs? The answer, most likely, is positive, and TDTs may play a key role to solve this puzzle.

## Paradigms and architectures

**Figure 1:**
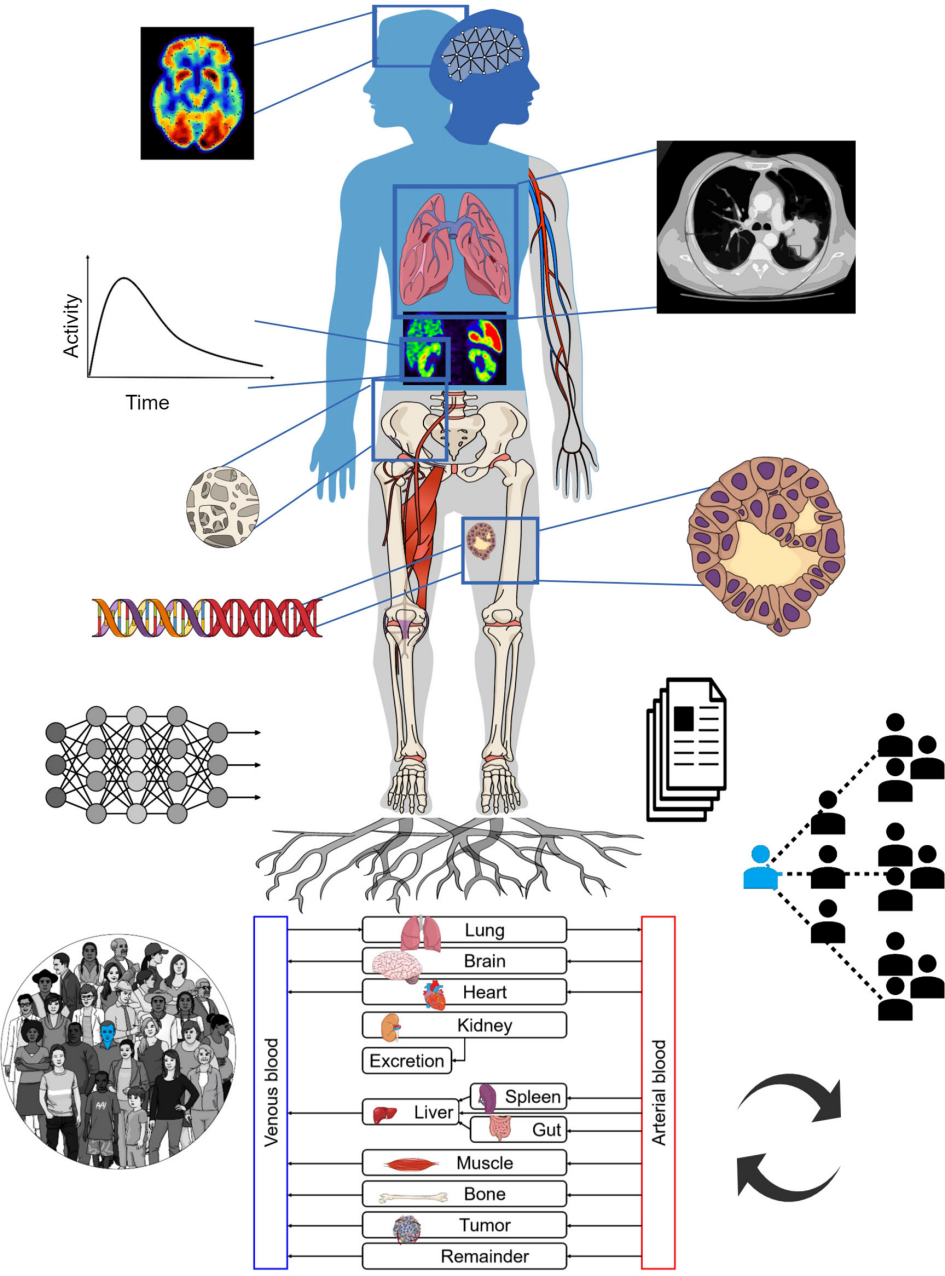
The TDT combines population-based knowledge with patient-specific information such as medical imaging, tumor genomics, etc. It can utilize physiologically based pharmacokinetic (PBPK) modeling towards individual therapy planning. AI may assist in different steps including data extraction and inverse methods for personalization of TDTs. The TDT can be updated with new collected data.

**Figure 1** depicts an overall view of TDTs. The TDT uses all available patient-specific information, including imaging data, documentation, tumor genomics, and combines this with knowledge derived from a population of patients. Physiologically based pharmacokinetic (PBPK) models can be developed based on theranostics, enabling simulation of different treatment scenarios (e.g. injected radioactivities) to help select optimal treatment scenarios for individual patients. AI can be used to assist in processing of the data, improving data quality and to solve inverse problems to personalize models to individual patients, in order to predict therapy responses. The TDT as such can be continuously updated with new data from the patient.

The TDT is much more than a specific solution. It is a discovery/solution-providing paradigm. It enables asking of numerous important questions and providing answers to them [e.g. (18, 19)]. It enables us to investigate a variety of intervention parameters that can be optimized; e.g. optimal injected radioactivities (for a given specific activity), sites of injection, injection intervals and profiles, and combination interventions/therapies (e.g. use of cardiovascular stress, blocking of receptors, radiosensitizers, immunomodulation, etc.). TDTs can help us ask important questions towards precision oncology for the individual patient and to look for reliable answers to those questions.

**Figure 2:**
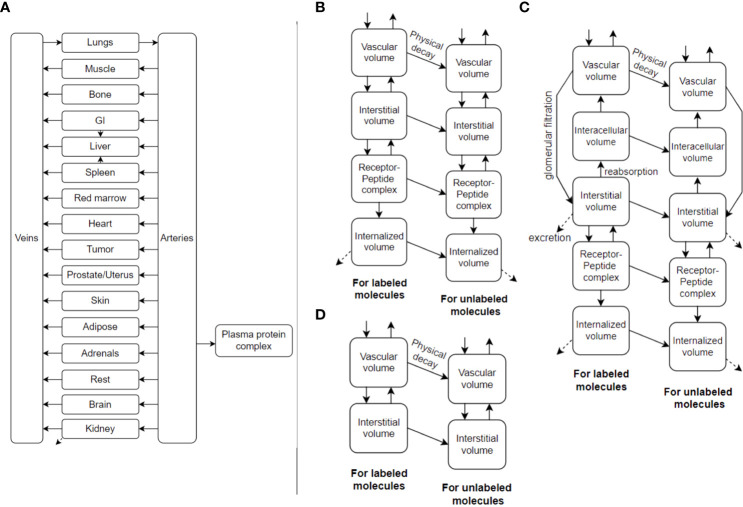
**(A)** An example PBPK model, utilizing ordinary differential equations (ODEs), which can also be called Physiologically-Based RadioPharmacoKinetic (PBRPK) Model. This model include organs **(B)** with and **(D)** without receptors, and includes **(C)** the kidney which has more sophisticated modeling. Radiopharmaceutical exchange (hot and cold components) between the different compartments is also represented.

TDTs can make significant use of PBPK models. An example is shown in **Figure 2**. PBPK models have been used in the past to gain understanding of important factors and optimal solutions at the population level (20–23), but also have the prospect of being personalized (18, 24) including use of AI methods to this end (i.e. inverse problems) (25). PBPK models commonly involve ordinary differential equations (ODEs), relating different compartments to one another. They can be stored and shared, for instance, in the XML format, and more specifically, SBML (system biology mark-up language) (26) format (e.g. see repository of models: https://www.ebi.ac.uk/biomodels).

PBPK models of the future may also involve other types of mathematical models such as spatiotemporal models (27). This includes partial differential equations (PDEs) having derivatives also with respect to space to better model spatial redistributions of radiopharmaceuticals within tumors and organs, as well as discrete and stochastic models (28), which are also very common in systems biology research. For optimal RPT planning, we likely have to go even further than conventional dosimetry. The limiting factor of administered activity is normal organ damage (18). Biologic events happen at the cell- and tissue-levels, thus *micro-scale* dosimetry is an important consideration and can be included in the TDT using macro- to micro-scale (multi-scale) modeling.

Building a digital twin should use the following criteria as a blueprint for any particular oncology applications: 1) What objectives does the digital twin have? 2) How much complexity is required? 3) Is there a suitable mechanism-based mathematical model? 4) Are the necessary data accessible or available? 5) Is it possible to characterize the uncertainties? The uncertainties can be in the observational data, model selection and parameter prediction steps (2). Uncertainties can emerge in (i) data acquisition, caused by experimental measurement errors, (ii) model selection, from assumptions and numerical techniques used to solve the mathematical models, and (iii) model parameters, due to intra- and inter-patient heterogeneities as well as the inherent stochasticity of tumor growth. To improve the accuracy of the modeled results, all these uncertainties must be taken into account both during model calibration and when interpreting predictions of digital twins (29).

## Discussion: Towards computational nuclear oncology

The goal of personalized RPTs is to tailor the treatment for each patient in order to maximize tumor control while minimizing critical damage to the organs at risk. In order to predict tumor response, we have to integrate dosimetry data with biological characteristics of the malignancy, including radiosensitivity, tumor heterogeneity and tumor microenvironment profile (particularly immune system activity). Similarly, normal organ toxicity is the interaction of deposited energy causing DNA damage (dosimetry) and biology of the organ (functional reserve, radiosensitivity, and repair capacity). The *paradigm* which integrates these puzzles is Computational Nuclear Oncology (CNO) and the *platform* which makes it possible is TDT.

Future of the field of theranostics is very bright if we stop taking a back-seat on the massive opportunities for personalization of therapies. We must bring together the fields of nuclear medicine, medical physics, dosimetry, radiobiology, multi-scale modeling, complex systems modeling, systems biology, computational medicine and AI to deliver optimal healthcare. The future includes use of advanced PET and SPECT imaging, e.g. long axial FOV PET scanners, combined with other imaging (e.g. CT or MR perfusion) and clinical data to enable generation of reliable TDTs, and predictive and personalized dosimetry, for optimal delivery of healthcare to our patients.

TDTs coupled with appropriate computational tools can be used for predictive absorbed radiation dose modeling; e.g. a model can be personalized based on pre- and/or intra-therapy molecular imaging. Different injection strategies and therapeutic intervals will be explored to improve delivered radiation dose to tumors while sparing healthy tissue. Corrective strategies such as adaptive dose planning, or adjuvant therapy with locoregional therapy (e.g. ablative therapy or external beam radiation therapy), or systemic chemotherapeutic strategies (e.g. immunotherapy or CAR-T therapy) can also be explored.

An example for direct application of TDTs would be in treatment of liver tumors using Y-90 microspheres for radioembolization. The intraarterial administration of the microspheres is simulated using Tc-99m macroaggregated albumin prior to the actual therapy to ensure a safe and efficient therapeutic application. SPECT/CT imaging of Tc-99m distribution within the patient’s liver and lung can be used to precisely plan the therapy and further to model and predict treatment response. This 3D distribution image from treatment simulation can update the TDT of the patient and the treatment decision between surgery, external radiation or systemic therapies can be carefully modeled and guide the final therapy plan.

Apart from personalized radioembolizations, we envision application of TDTs in RPTs; e.g. Lu-177-PSMA therapy of advanced prostate cancer. Pre-therapeutic F-18-PSMA or Ga-68-PSMA PET/CT images will be adopted in TDTs with PBPK modelling to generate individualized therapy plans for patients including the therapeutic activity, number of therapy cycles, time between cycles and more parameters.

Image-guided digital twins for clinical oncology face a number of challenges: (i) Current mathematical models may have limited modeling of biological processes at different-scales thus there is a need for improved multi-scale modeling. (ii) Only a small portion of the pertinent cancer biology is examined by common imaging modalities. (iii) Computational techniques such as finite element methods (FEM) and finite difference methods (FDM) have limitations related to geometric discretization. The use of hybrid AI-mechanistic approaches built as neural network encoding may be able to entangle these restrictions. (iv) Model validation, model selection, and uncertainty quantification are other limitations which need to be tackled, and present important frontiers.

It is important to remember, as mentioned in the introduction, that uncertainties in absorbed doses by organs-at-risk (max:min) span an order of magnitude in current non-personalized RPT practice (16, 17). TDTs will not have zero uncertainties, but are expected to significantly reduce these uncertainties, and to act as powerful tools for clinical treatment decisions. All in all, this will provide patients with truly personalized treatment decisions towards optimizing therapeutic outcomes.

The future is bright if we day-dream, do what is right, and open ourselves to the massive opportunities that enable better care for our patients.

## Data availability statement

The original contributions presented in the study are included in the article/Supplementary Material. Further inquiries can be directed to the corresponding author.

## Author contributions

AR contributed to key ideas and wrote main parts of manuscript. JB-L contributed to key ideas, generated **Figure 1** and contributed to improve the manuscript. AF-P contributed to key ideas, generated **Figure 2** and contributed to improve the manuscript. FY, MS and CU contributed to key ideas and to improve the manuscript. BS conceived the original TDT idea, contributed to key discussions and to improve the manuscript. All authors contributed to the article and approved the submitted version.
